# Biosynthesized BiFe_2_O_4_@Ag nanoparticles mediated *Scenedesmus obliquus* induce apoptosis in AGS gastric cancer cell line

**DOI:** 10.1038/s41598-024-57157-0

**Published:** 2024-05-04

**Authors:** Hossein Shamsi, Reza Yari, Ali Salehzadeh

**Affiliations:** 1 Department of Biology, Rasht Branch, Islamic Azad University, Rasht, Iran; 2 Department of Biology, Borujerd Branch, Islamic Azad University, Borujerd, Iran

**Keywords:** AGS cell line, Apoptosis, BiFe_2_O_4_@Ag nanoparticles, Cell cycle, Flow cytometry, Biological techniques, Cancer, Cell biology, Chemistry

## Abstract

The use of magnetic metal nanoparticles has been considered in cancer treatment studies. In this study, BiFe_2_O_4_@Ag nanoparticles were synthesized biologically by *Scenedesmus obliquus* for the first time and their anticancer mechanism in a gastric cancer cell line was characterized. The physicochemical properties of the nanoparticles were evaluated by fourier transform infrared spectroscopy (FT-IR), X-ray diffraction (XRD), energy-dispersive X-ray spectroscopy (EDS), scanning electron microscopy (SEM), transmission electron microscopy (TEM), dynamic Light Scattering (DLS), and zeta potential analyses. Cell viability and nuclear damage were investigated by the MTT and Hoechst staining assays, respectively. Flow cytometry analysis was performed to determine the frequency of the necrotic and apoptotic cells as well as cell cycle analysis of the nanoparticles-treated cells. Physicochemical characterization showed that the synthesized particles were spherical, without impurities, in a size range of 38–83 nm, with DLS size and zeta potential of 295.7 nm and -27.7 mV, respectively. BiFe_2_O_4_@Ag nanoparticles were considerably more toxic for the gastric cancer cells (AGS cell line) than HEK293 normal cells with IC_50_ of 67 and 117 µg/ml, respectively. Treatment of AGS cells with the nanoparticles led to a remarkable increase in the percentage of late apoptosis (38.5 folds) and cell necrosis (13.4 folds) and caused cell cycle arrest, mainly at the S phase. Also, nuclear fragmentation and apoptotic bodies were observed in the gastric cancer cells treated with the nanoparticles. This study represents BiFe_2_O_4_@Ag as a novel anticancer candidate against gastric cancer that can induce cell apoptosis through DNA damage and inhibition of cell cycle progression.

## Introduction

Gastric cancer is known as one of the most common types of cancer in many parts of the world. The increase in the number of incidences and mortality by this disease has raised serious health concerns^1^. Current treatment methods include surgery to remove the cancerous tissues and the use of chemotherapy drugs. However, current treatments lack sufficient efficacy in advanced disease cases and metastatic types. This has led researchers to study the field of designing and testing novel and effective drugs against this disease.

Use of metal nanoparticles has been proposed as a new approach in cancer treatment studies. Many metal elements, when synthesized on a nano-scale, may gain new properties such as increased reactivity and improved permeability, which can be used in various fields^2,3^. However, the use of such compounds in biomedical fields is not always successful. The clinical use of such compounds faces limitations due to their potential toxic effects or low efficacy^4^. In addition, due to their small size, they can be distributed and accumulated in non-target tissues and cause unwanted side effects. Therefore, the design and application of multifunctional nanocomposites can be considered a leading step in the field of using metal nanoparticles in biomedical applications. In this approach, several nanoparticles can be conjugated to provide a more efficient and less toxic composite. The use of magnetic nanoparticles, such as iron oxide nanoparticles, in the fabrication of such composites can provide directed delivery to the target tissues using an external magnetic force, and in this way, in addition to improving the effectiveness of the nanocomposite, it may reduce drug toxicity by reducing the dispersion and accumulation of particles in non-target tissues^5^. However, the level of toxicity for healthy cells, biodistribution, stability, immune responses and clearance are among the most important challenges that must be considered in the clinical application of metal nanocomposites.

Biological synthesis is another approach to obtaining less toxic metal nanoparticles to be used in biomedical fields. Unlike physicochemical methods, which use toxic substances and harsh conditions for the synthesis of nanoparticles, in the biological method, living organisms or their products are used in the synthesis of nanoparticles^6,7^. Algae are a group of autotropic organisms with economic and ecological importance. Algae are used for the synthesis of nanoparticles as they have a high potential to accumulate metal, are easy to handle and cultivate, can grow at low temperatures, and are less toxic to the environment. The use of algae is mainly due to their high capacity to take in metals and reduce metal ions, relatively low production costs, and most importantly their ability to produce nanoparticles at a large scale^8^. The use of algae extracts is among the most common methods of biological synthesis of metal nanoparticles. *Scenedesmus obliquus* is an algal species that has shown great potential to be used in various fields of biotechnology, including the removal of chemical pollutants and biofuel production. In addition, recent studies have shown that the *S. obliquus* extract can be used to synthesize metal nanoparticles^9^.

Bismuth complexes have shown medicinal properties, including antifungal, antibacterial, and anticancer activities^10^. Recent studies have shown that bismuth nanoparticles can prevent the proliferation of cancer cells by inducing the production of reactive oxygen species and the generation of oxidative stress^11,12^. In a previous work, Hernandez-Delgadillo et al.,^13^ reported that bismuth nanoparticles induced dose-dependent growth inhibition of breast cancer cells and found that breast cancer cells were more vulnerable than healthy breast cells. They found that bismuth nanoparticles reduce plasma membrane integrity and exert genotoxic effects on breast cancer cells. In addition, Ahamed et al.,^11^ found that exposure to Bismuth oxide nanoparticles generates oxidative stress in breast cancer cells, leading to increased lipid peroxidation, GSH depletion, and reduced SOD activity. Furthermore, the nanoparticles caused an apoptotic response in treated cells that were associated with impaired regulation of *Bcl-2*, *Bax,* and *caspase-3* genes. Furthermore, silver nanoparticles are known as biocompatible and effective anticancer agents against various cancer cells^14,15^. The anticancer mechanism of silver nanoparticles mainly depends on the alteration of cellular redox status which can damage cellular structures such as cytoplasmic membrane and nucleic acid, which results in proliferation inhibition^17–19^.

Considering the magnetic properties of Fe_2_O_4_ and the anticancer potential of bismuth and silver nanoparticles, in this work, BiFe_2_O_4_@Ag nanoparticles were synthesized by *S. obliquus* extract for the first time and their anticancer potential in a gastric cancer cell line was investigated.

## Materials and methods

### Preparation of S. obliquus extract

The *S. obliquus* extract was prepared according to the method described by Salehzadeh et al.^19^. To prepare the aqueous extract, 1 g of *S. obliquus* (lyophilized) was added to 50 mL of dH_2_O and preserved in incubator at 55 °C for 30 min. Next, the extract was centrifuged at 5000 rpm for 10 min (Kaida, China) and the supernatant was passed through a filter paper. The supernatant was preserved at 4 °C for constructing of BiFe_2_O_4_@Ag.

### Nanoparticle synthesis

BiFe_2_O_4_ nanoparticles were synthesized according to the method reported by Salehzadeh et al.^19^. At first, 0.2 g of Bi(NO_3_)_3_ (5mM) and 0.4 g of Fe(NO_3_)_3_.9H2O (10mM) were dissolved in 100 ml of distilled water and the reaction mixture was stirred for 60 min in a water bath at 80 °C. Next, NaOH solution (6 M) was gradually added to the suspension. The suspension was centrifuged and BiFe_2_O_4_ nanoparticles were harvested, washed, and dried at 70 °C.

In the next step, 40 mg of BiFe_2_O_4_ nanoparticles and 20 mg of silver nitrate were added to distilled water and the resulting mixture was stirred for 50 min at 55 °C. Then, the *S. obliquus* extract was added to the reaction mixture and stirred overnight at room temperature. Finally, the nanoparticles were separated by centrifugation, washed with ethanol and distilled water, and dried at 70 °C.

### Physico-chemical characterization of nanoparticles

Fourier transform infrared spectroscopy (FT-IR)^20,21^, X-ray diffraction (XRD)^22^, scanning electron microscopy (SEM)^23,24^ and transmission electron microscopy (TEM)^26–28^, Energy-dispersive X-ray spectroscopy (EDS), zeta potential, and Dynamic Light Scattering (DLS) analyses were used to characterize the physical–chemical properties of the nanoparticles. The functional groups of BiFe_2_O_4_ and BiFe_2_O_4_@Ag nanoparticles were investigated using FT-IR analysis by a Nicolet IR 100 spectrophotometer (400–4000 cm^−1^). UV visible spectroscopy was done in the range of 200–800 nm (PerkinElmer, LAMBDA 1050). The crystal structure of the BiFe_2_O_4_@Ag nanoparticle was investigated using a Philips X'Pert MPD diffractometer (λ = 1.54056 Å) (Cu-Kα X-ray tube). The morphologic feature and size of the particles were determined by SEM (TESCAN MIRA3, Czech Republic) and TEM (Zeiss-EM10C-100 kV, Germany) microscopes. EDS assay was done to determine the elemental composition of the nanoparticles (TESCAN Mira3). Furthermore, DLS and zeta potential analyses were performed to determine the hydrodynamic particle size and surface charge of the particles using a Malvern Instruments Ltd, 6.32 device.

### Cell culture

Cancer (AGS) and normal (HEK293) cell lines were purchased from the Pasteur Institute of Iran, as gastric cancer and normal human fibroblast cells, respectively. Cell lines were cultured in RPMI 1640 medium (supplemented with 10% FBS, 100 IU/mL penicillin, 100 μg/mL streptomycin, and 2 mM glutamine) under standard conditions (37 °C, in humidified air containing 5% CO_2_).

### Cell viability assay

Cytotoxicity of the BiFe_2_O_4_@Ag nanoparticles for gastric cancer and normal cell lines was assessed by MTT assay^29–31^. In brief, about 10,000 cells were grown in 96-well plates to reach the 50% confluency and were treated with a concentration gradient (0–500 µg/ml) of the nanoparticles for 24 h. Then, the medium was aspirated, 200 µl of the MTT (2-(4,5-dimethythiazol-2-yl)-2,5-diphenyltetrazolium bromide) solution was added to the wells, and incubated for four hours. In the next step, the contents of the wells were emptied and 200 µl of DMSO was added and re-incubated for 30 min at room temperature. Finally, the optical absorbance of the wells was measured at 570 nm by a Bio-Rad microplate reader. The inhibitory percentage of the nanoparticles for the studied cell lines was measured using the following formula^30^. The inhibitory effect of Cisplatin, as a standard anticancer drug, for the AGS cell line was also determined.

$$Inhibition (\mathrm{\%})=\frac{Abs\,of\,control-Abs\,of\,Test}{Abs\,of\,control}\times 100$$

### Flow cytometry assay

Flow cytometry assay was used to study the effect of BiFe_2_O_4_@Ag nanoparticles on the percentage of healthy, apoptotic, and necrotic cells in AGS cell line. AGS cells were propagated and then, 5 × 10^5^ of cells treated with BiFe_2_O_4_@Ag nanoparticles at their 50% inhibitory concentration (IC_50_), while control cells were treated with PBS for 24 h. Thereafter, the cells were washed two times with phosphate-buffered saline and resuspended in binding buffer. Then, the propidium iodide and Annexin V (Roche, Germany) dye was added to cells and incubated for 15 min in dark condition^28^. Finally, the percentage of healthy, apoptotic, and necrotic cells was measured by the ParTec™ flow cytometry instrument (Germany).

### Cell cycle analysis

In order to investigate the changes in the cell content, cell cycle analysis was accomplished by flow cytometry method in cells treated with BiFe_2_O_4_@Ag nanoparticles. According to procedure of Sigma-Aldrich (USA) Cell Cycle Analysis Kit, the AGS cells were cultured with a density of 5 × 10^5^ in a 6-well plate and subjected to 67 μg/mL of BiFe_2_O_4_@Ag nanoparticles for 24 h in a CO_2_ incubator***.*** In following step, the cells were collected and washed with PBS buffer*.* Afterward, cold ethanol (70%) was used for fixation, and propidium iodide was used to stain of the cells. Further, the cells were treated with RNase A (100 µg/mL). In the final step, a flow cytometer (ParTec™, Germany) was used to measure the DNA content of the cells.

### Hoechst staining

Hoechst staining assay was performed to investigate the nuclear damages caused by BiFe_2_O_4_@Ag nanoparticles in AGS cells. The AGS cells were grown in 6-well plates with a density of 5 × 10^5^ for 24 h and exposed to 67 μg/mL of BiFe_2_O_4_@Ag nanoparticles. Subsequent to washing with PBS, the cells were fixed by 4% formaldehyde and then, incubated with Hoechst 33258 (5 μg/ml) at 37 °C for 4h in darkness. Untreated cells were regarded as the control. After washing the cell monolayer with PBS, the cells were examined under a fluorescent microscope (Zeiss, Wetzlar, Germany)^30^.

### Statistical analyses

Statistical analyses were performed by the SPSS. 16.0 software. *one-way ANOVA* analysis was used to evaluate significant differences and the p-value < 0.05 was regarded as statistically significant^31,32^.

## Results

### Physicochemical characteristics

**Figure 1:**
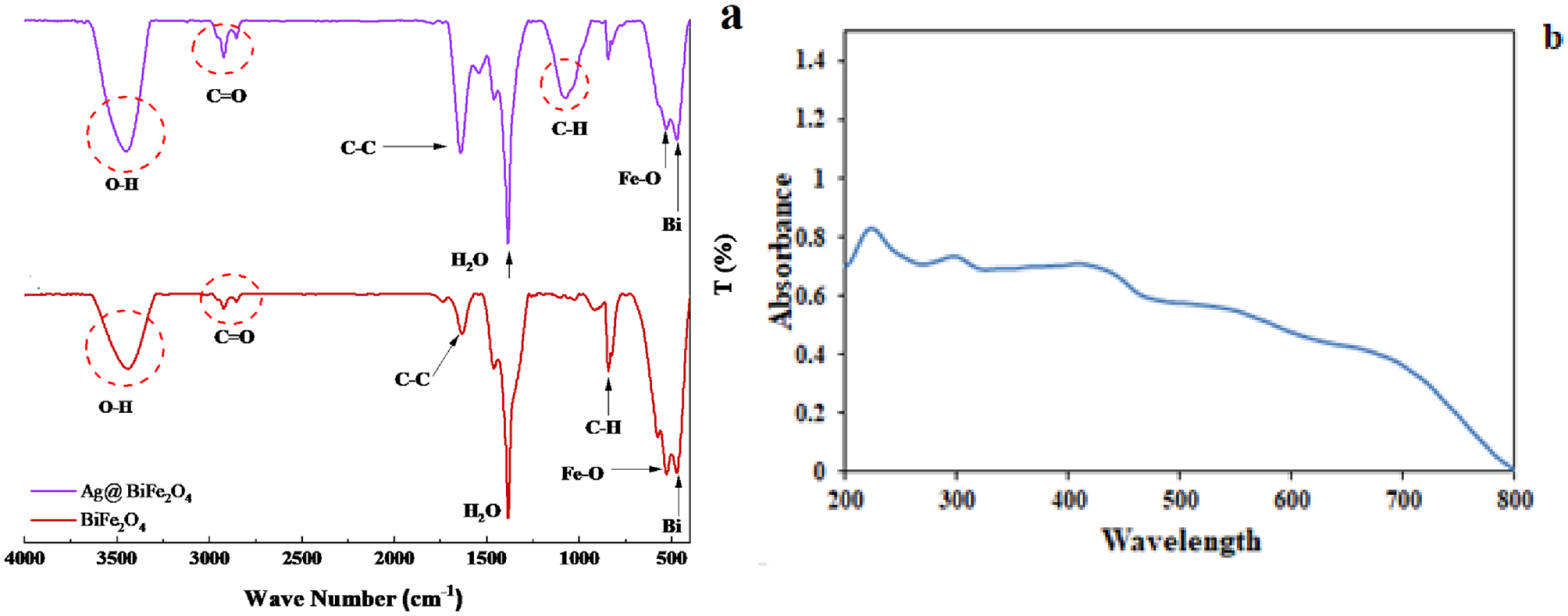
(**a**) FT-IR analysis of BiFe_2_O_4_ and BiFe_2_O_4_@Ag nanoparticles. (**b**,**c**) UV visible spectroscopy of BiFe_2_O_4_@Ag nanoparticles. Comparing the spectra confirms the formation of the particles.

According to the FT-IR spectrum of BiFe_2_O_4_, some vibrations associated with the metal oxide (M–O) bonds can be observed at 474 and 526 cm^−1^, and the peak at 825 cm^−1^ is related to the C–H bond. Furthermore, two peaks at 1323, and 1383 cm^−1^, could be related to the stretching vibrations of C-H and N–O bonds. The vibrations associated with the O–H bond can be observed at 3449 cm^−1^. Characterization of the functional groups of BiFe_2_O_4_@Ag showed several peaks related to the M–O bond at 227 and 530 cm^−1^, and stretching bonds at 823, 1079, and 1389 cm^−1^ that are associated with C–H, C–O, and C–H bonds, respectively. The stretching vibration of the C=O bond can be identified at 1648 cm^−1^. In addition, some vibrations at 2925 and 3444cm^−1^ are observed which are due to the O–H bonds of the adsorbed water molecules. The results were displayed in Fig. 1.

**Figure 2:**
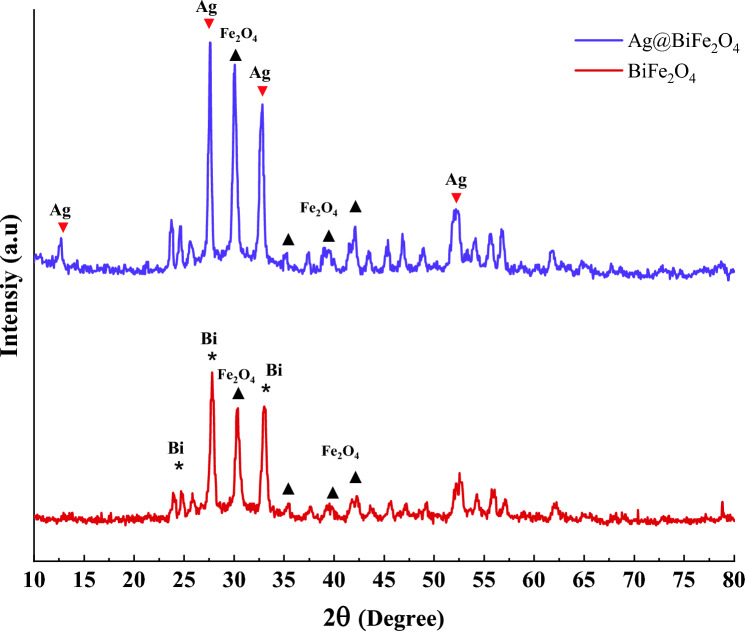
XRD analysis of BiFe_2_O_4_ and BiFe_2_O_4_@Ag nanoparticles.

According to the XRD assay, the peaks at 2θ of 27, 33, and 53 degrees are associated with the Ag atoms, which is in agreement with the JCPDS card No. 89-3722^33^. The peak at 2θ of 25 degrees is related to the bismuth atoms^34^, and the peaks associated with Fe_2_O_4_ can be found at 30, 35, 42, 45, and 62 degrees^35^ (Fig. 2).

**Figure 3:**
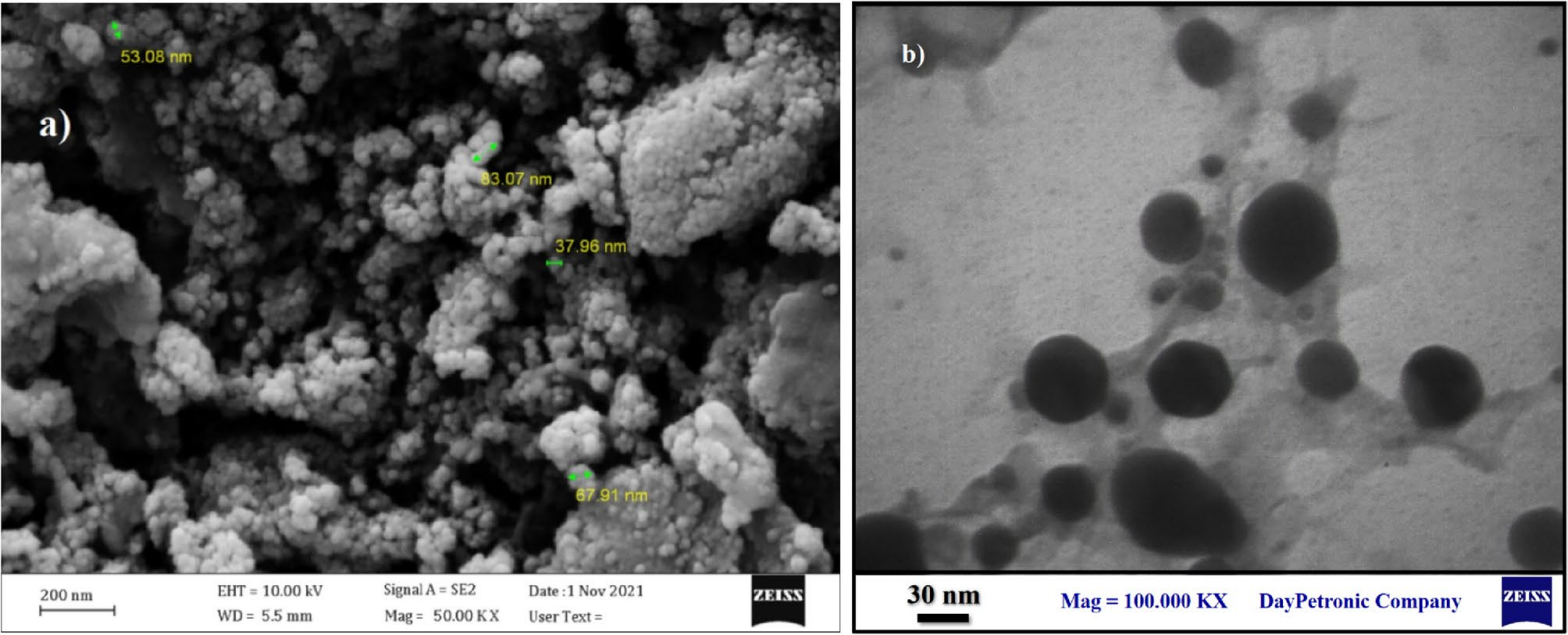
(**a**) SEM and (**b**) TEM images of BiFe_2_O_4_@Ag nanoparticles. The particles are spherical and in a size range of 38–83 nm.

**Figure 4:**
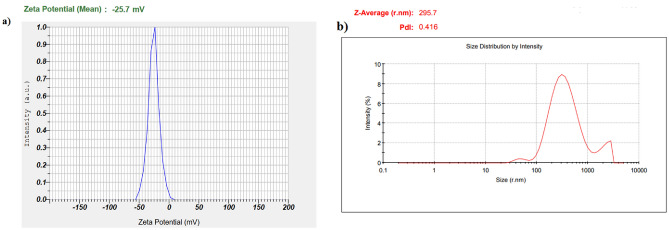
(**a**) Zeta potential and (**b**) DLS size of BiFe_2_O_4_@Ag nanoparticles. The surface charge and size of the particles in an aqueous environment were − 27.7 mV and 295.7 nm, respectively.

**Figure 5:**
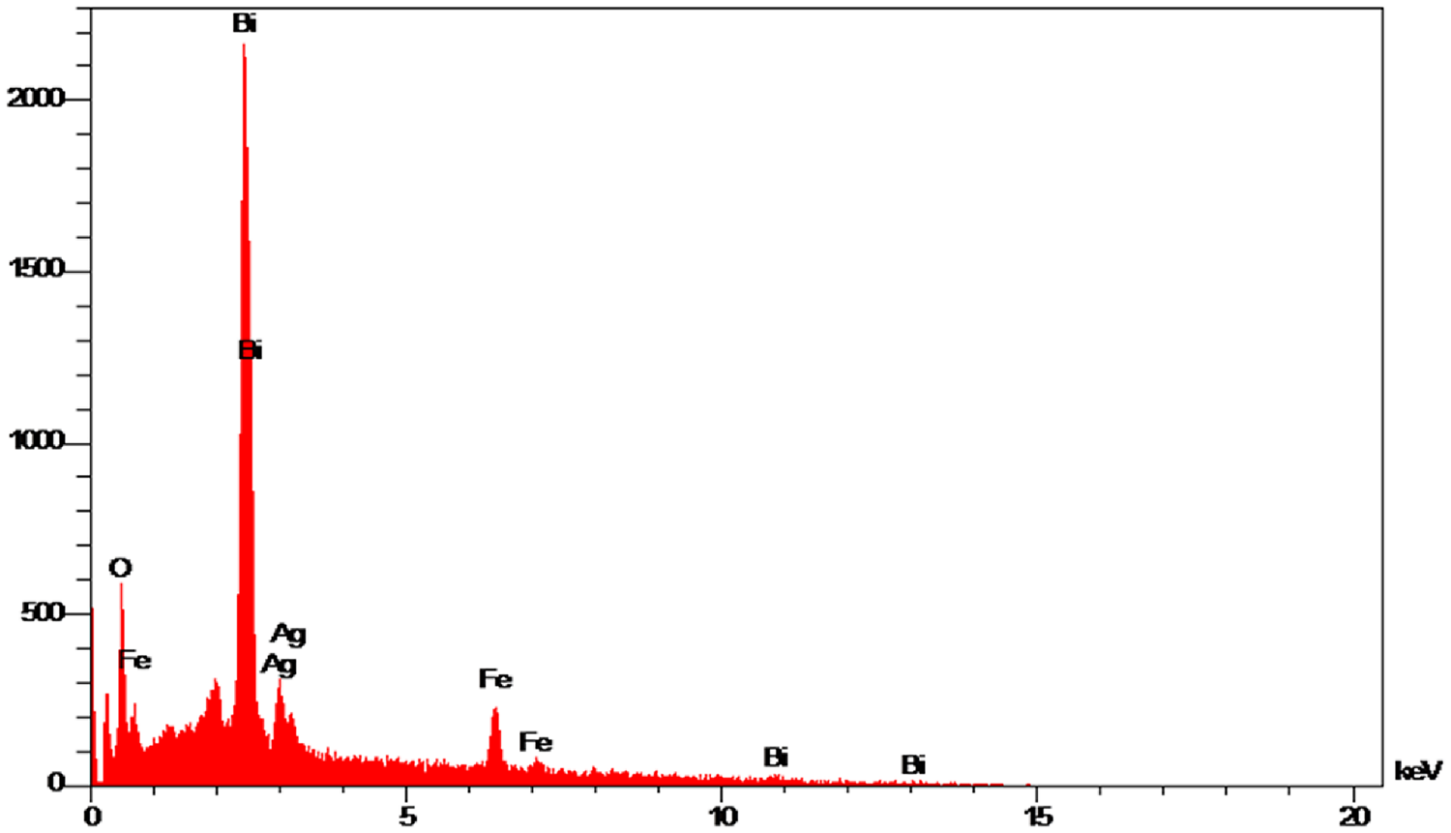
EDS of BiFe_2_O_4_@Ag nanoparticles. The particles contained Ag, C, O, Fe, and Bi atoms, and elemental impurity was not found.

According to the electron microscope imaging, the synthesized nanoparticles were spherical and within a size range of 38–83 nm. Figure 3 displays the SEM and TEM images of the BiFe_2_O_4_@Ag nanoparticles. In addition, the zeta potential and DLS size of the particles were − 27.7 mV and 295.7 nm (Fig. 4). Characterization of the elemental composition of the BiFe_2_O_4_@Ag nanoparticles showed that the particles were made of O, Fe, Bi, and Ag atoms and had no elemental impurities. The results were presented in Fig. 5.

### MTT assay

**Figure 6:**
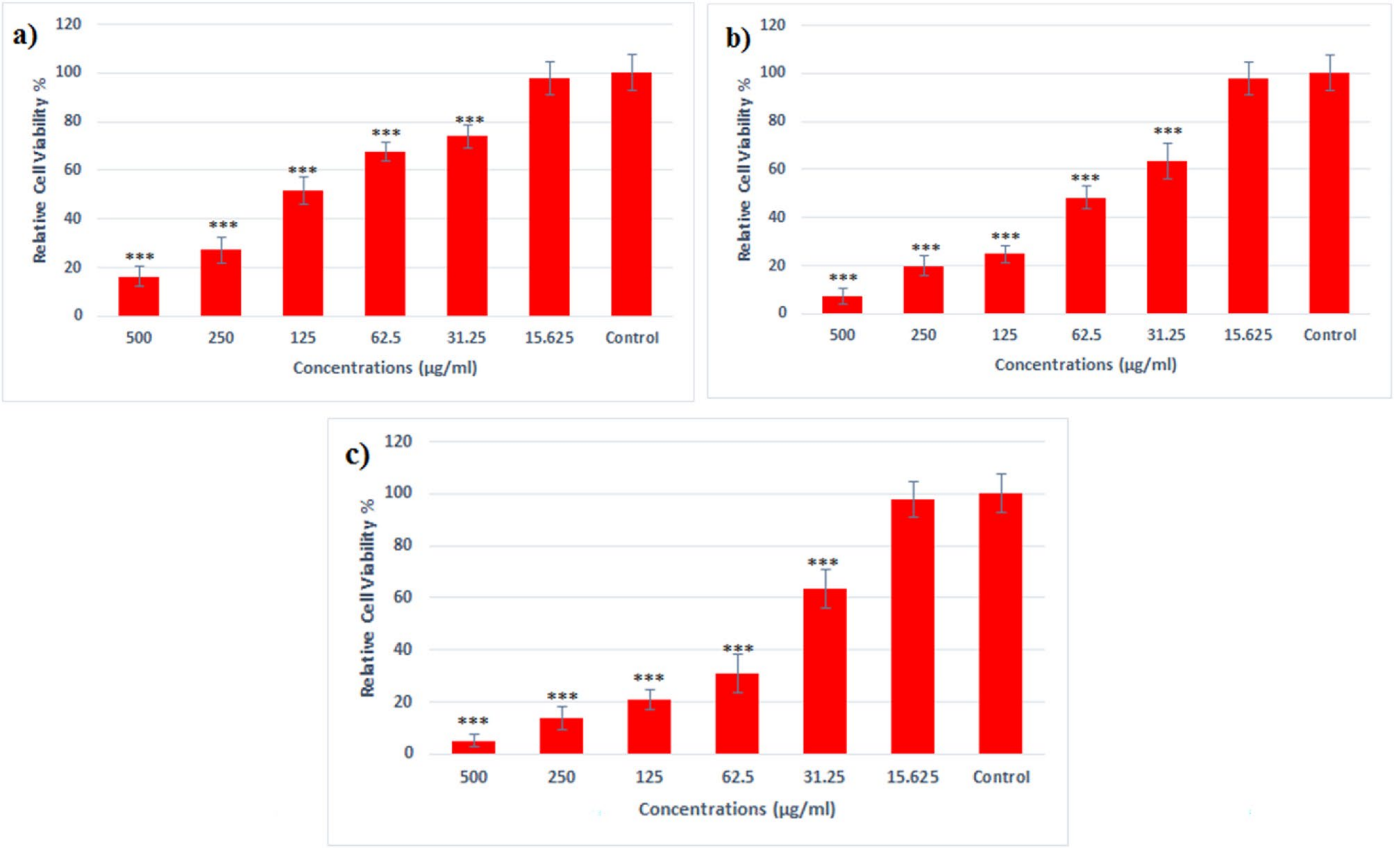
Viability assay for (**a**) HEK293 cells and (**b**) AGS cells after treatment with BiFe_2_O_4_@Ag nanoparticles. (**c**) Cell viability of AGS cells after treatment with cisplatin. Cancer cells were considerably more susceptible to the nanoparticles than normal cells. The IC_50_ of BiFe_2_O_4_@Ag nanoparticles for the normal and cancer cells were 117 and 67 µg/ml, and the IC_50_ of cisplatin for cancer cells was 55 µg/ml. (***) indicates significant differences at p < 0.001.

The viability of gastric cancer cells and normal human cells after treatment with different concentrations of BiFe_2_O_4_@Ag nanoparticles was investigated. Our results showed that, although the nanoparticles had dose-dependent toxicity for both cell lines, it was considerably more toxic for the cancer cells than normal ones. The 50% inhibitory concentration (IC_50_) of the nanoparticles for the AGS and HEK293 cell lines were 67 and 117 µg/ml, respectively. Furthermore, the IC_50_ of cisplatin for AGS cells was 55µg/ml. The results were displayed in Fig. 6.

**Figure 7:**
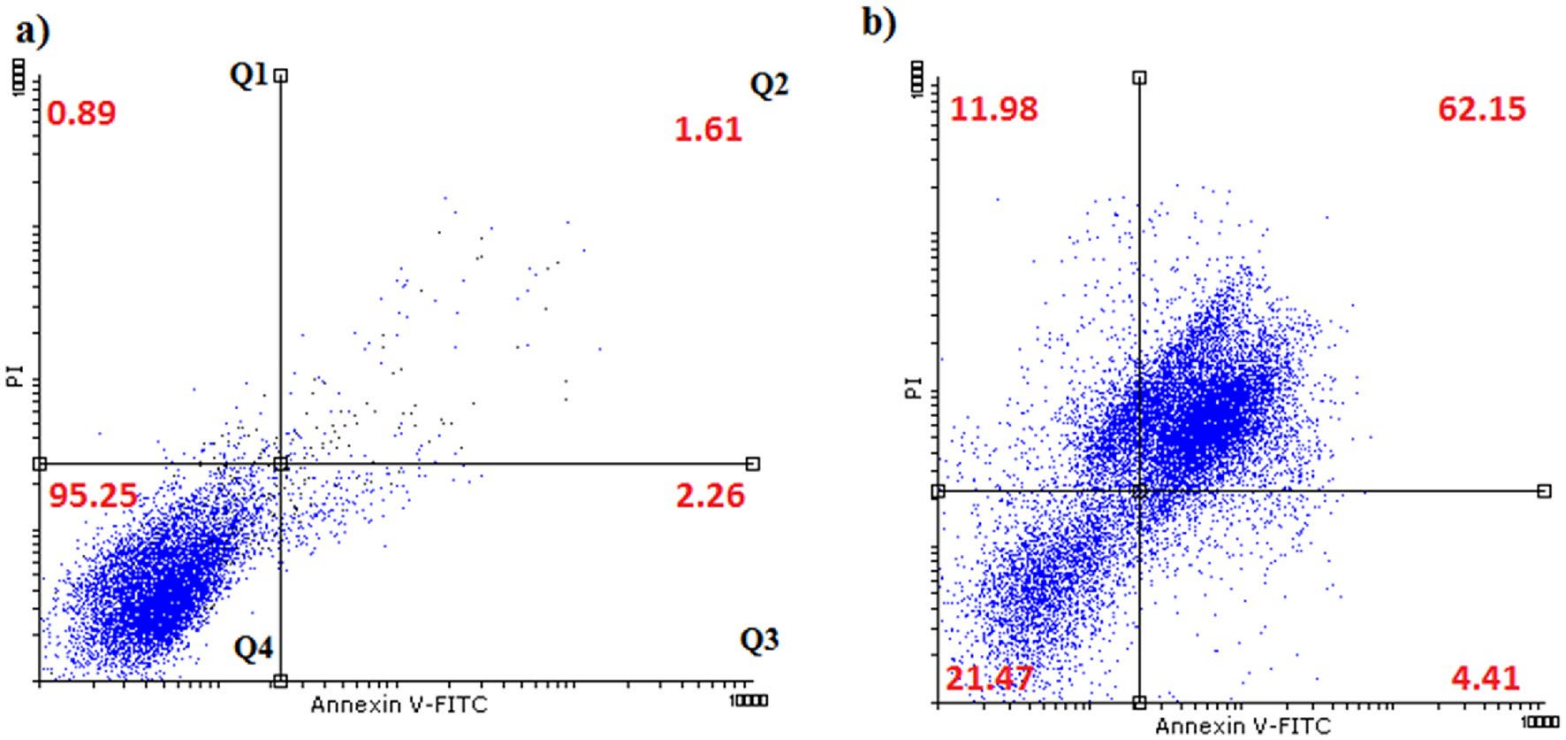
Flow cytometry analysis of AGS cells treated with BiFe_2_O_4_@Ag nanoparticles. treatment with the nanoparticles significantly increases the population of cell necrosis, primary and late apoptosis. Q1: necrotic cells, Q2: late apoptosis, Q3: primary apoptosis and Q4: live cells.

According to the flow cytometry assay, treating gastric cancer cells with BiFe_2_O_4_@Ag nanoparticles led to a remarkable increase in the population of cell necrosis and apoptosis. The highest increase was observed for late apoptosis, which increased from 1.61 to 62.1%, after treatment with the nanoparticles. Furthermore, the percentage of necrotic cells increased from 0.89 to 11.98% (Fig. 7).

### Cell cycle analysis

**Figure 8:**
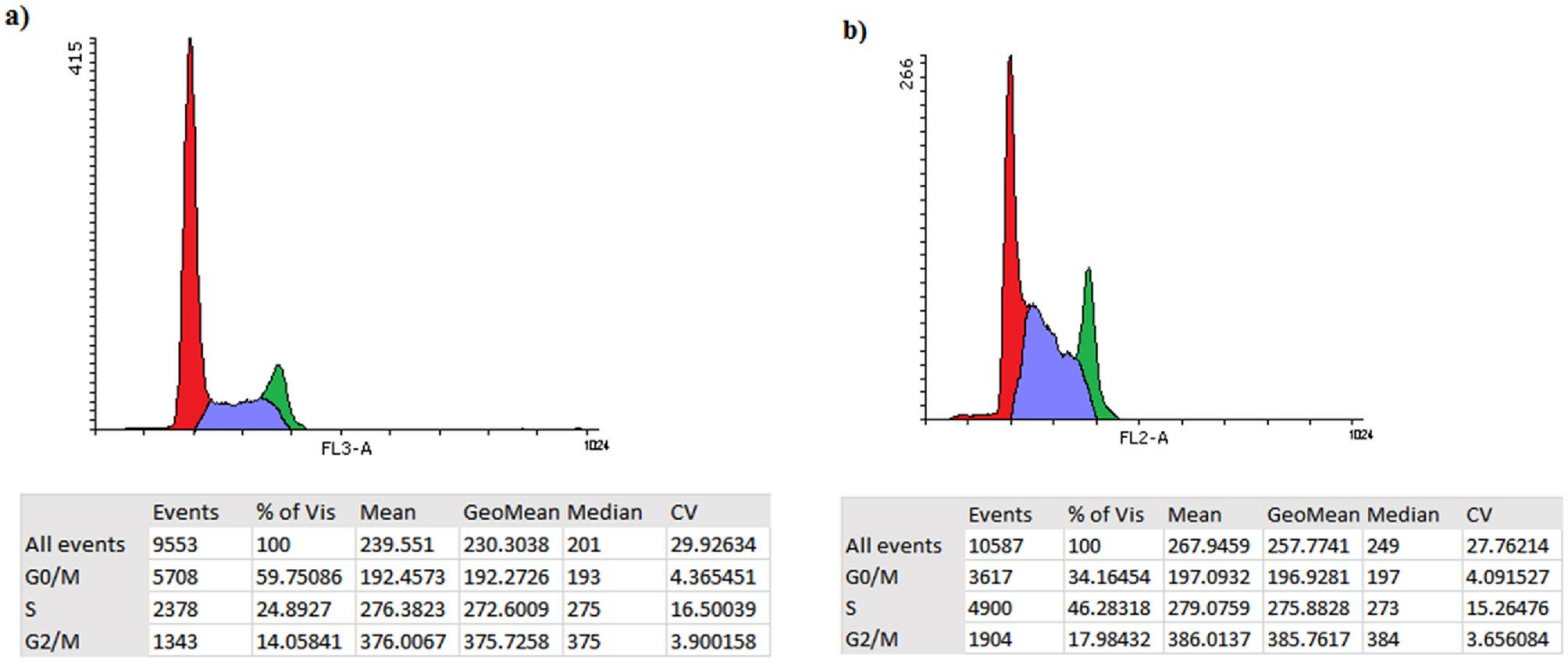
Cell cycle analysis of AGS cells. (**a**) Control, (**b**) treated with BiFe_2_O_4_@Ag nanoparticles. Upon treatment with the BiFe_2_O_4_@Ag nanoparticles, the frequency of the cells at the G0/M phase was reduced significantly while the population of the cells arrested at the S phase significantly increased.

According to the results, upon treatment with the BiFe_2_O_4_@Ag nanoparticles, the frequency of the AGS cells at the G0/M phase was reduced by 25.6%. In contrast, the frequency of the cells arrested at the S phase significantly increased from 24.9 to 46.3% (+ 21.4%). Also, the population of the cells at the G2/M phase was slightly increased after treatment with the nanoparticles (Fig. 8).

### Nuclear damage

**Figure 9:**
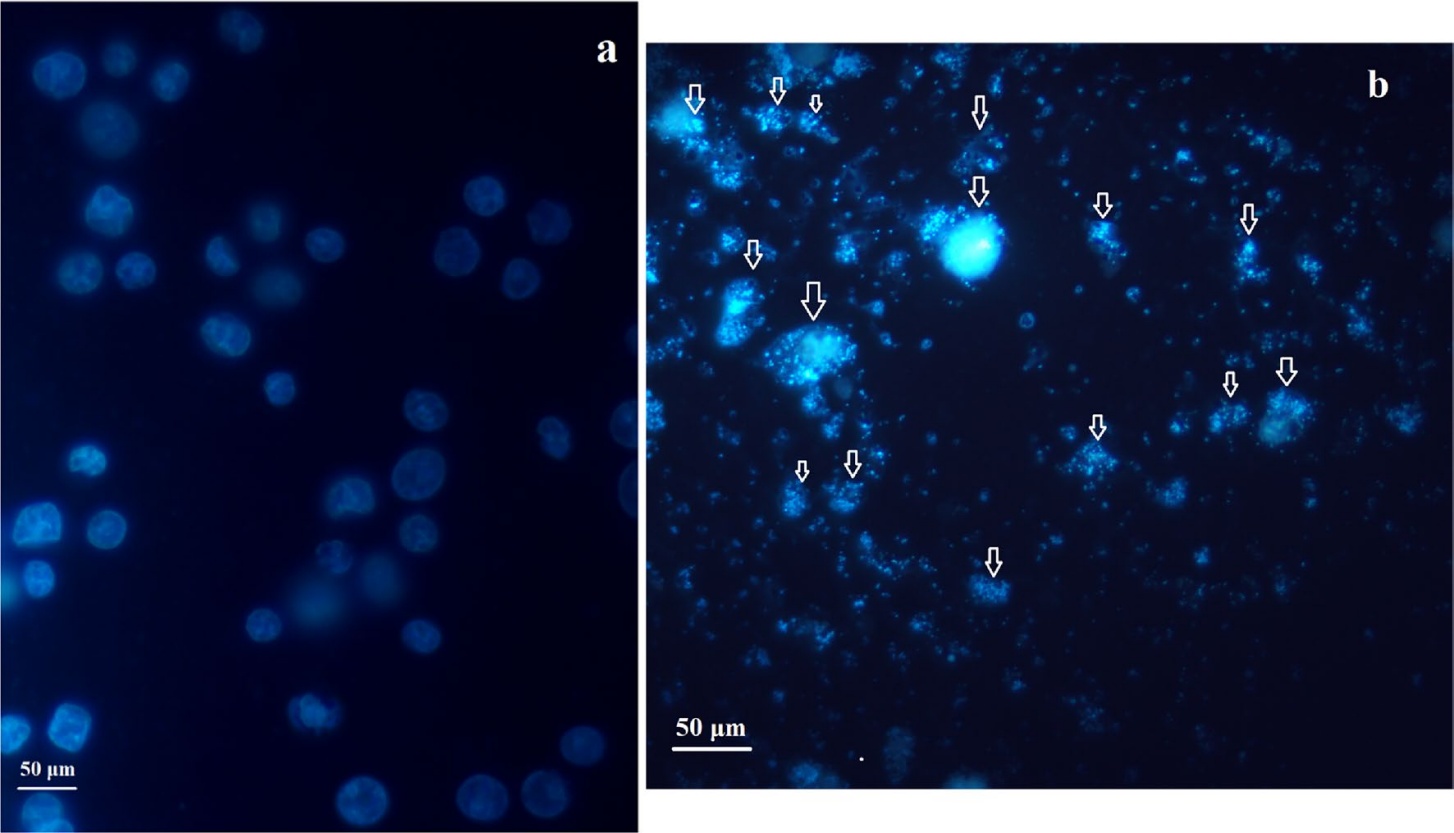
Hoechst staining of AGS cells. (**a**) Control, (**b**) BiFe_2_O_4_@Ag nanoparticles-treated cells. Fragmentation of the cell chromatin, formation of apoptotic bodies, and chromatin condensation were the main observed changes in treated cells.

According to the Hoechst staining assay, after treatment of the AGS cells with BiFe_2_O_4_@Ag, some morphological changes favoring apoptosis induction were observed. The main changes include fragmentation of the cell chromatin, formation of apoptotic bodies, as well as condensation of cell chromatin. The results were displayed in Fig. 9.

## Discussion

Combination therapies using metallic and magnetic nanoparticles have provided new insight into cancer treatment studies. In this approach, different nanoparticles are combined and manipulated to obtain a nanocomposite with improved efficacy and biocompatibility and less toxicity^36^. Due to the anticancer potential of silver and bismuth nanoparticles, and the magnetic properties of iron oxide nanoparticles, this work was conducted to synthesize BiFe_2_O_4_@Ag nanoparticles and investigate its effect on a gastric cancer cell line.

Physicochemical characterization of the nanoparticles showed that the particles were synthesized in the nano-scale size range. Nano-size particles have generally large surface areas which can increase their reactivity. Also, due to their small size, they can easily penetrate the host tissues and exert their effects on the target cells. Furthermore, the surface charge of the particles was -27.7 mV which provides enough repulsive force between the particles to avoid particle agglomeration^37^.

Based on the MTT results, BiFe_2_O_4_@Ag significantly inhibited the growth of the AGS cells. In addition, it had remarkably higher toxicity for the gastric cancer cells than the human normal cell line. The cytotoxic effect of the BiFe_2_O_4_@Ag nanoparticles seems to be mainly associated with its silver and bismuth ions. Many studies have reported the inhibitory effects of silver nanoparticles on various cancer cell lines^40–42^. Similar to our work, Mousavi et al.^40^ reported dose-dependent toxicity of silver nanoparticles for AGS cell line which considerably inhibited cell proliferation and caused cell apoptosis. Furthermore, it has been reported that bismuth nanoparticles have higher cytotoxicity for cancer cells than healthy cells which is in agreement with our work. It was understood that bismuth nanoparticles can interrupt cytoplasmic membrane integrity and cause DNA damage, leading to cell death^13^.

The inhibitory effect of silver nanoparticles on cancer cells is mainly related to the overproduction of ROS molecules as well as its direct interaction with biological molecules. Previous studies have shown that silver can cause an excessive increase in ROS levels and oxidative stress, which leads to damage to the vital parts of cancer cells, such as the cytoplasmic membrane, DNA molecule, and cellular enzymes, thus inducing apoptosis and cell death^41,42^. In addition, the increased generation of ROS can modulate signaling pathways such as the mitogen-activated protein kinase (MAPK), phosphoinositide 3- kinase (PI3k)/Akt, and the p53 pathways, leading to cell cycle arrest and apoptosis induction^43,44^. The generation of oxidative stress in bismuth-treated cells has been reported in the literature^11,12^. Therefore, damage to cell components by overproduction of ROS molecules seems to be the major cytotoxic mechanism of BiFe_2_O_4_@Ag nanoparticles in gastric cancer cells.

In addition to oxidative stress, metal nanoparticles can interfere with cell signaling pathways leading to inhibition of cell cycle progression and apoptosis induction. For example, it was found that silver nanoparticles can exert cytotoxic effects on mammalian cells through inhibition of endoplasmic reticulum (ER). The ER functions in protein folding and assembly, lipid biosynthesis and cellular calcium storage. Due to the critical role of ER in cell homeostasis, dysfunction of ER could result in cellular damage and apoptosis. In other words, perturbation of the ER function, which is called ER stress, is associated with various cellular damage, leading to ER-induced cell apoptosis and cell death^45^.

The difference in the susceptibility of normal and cancer cells to the nanoparticles can be related to the difference in their metabolic status and proliferation rate. Cancer cells are naturally under higher oxidative stress compared to normal cells due to higher metabolic and hyperproliferation which cause increased ROS generation^46,47^. For this reason, their tolerance against the excessive production of oxidative radicals is lower than normal cells and their antioxidant systems are less efficient in detoxifying oxidative molecules. Therefore, these cells can be more susceptible to the studied nanoparticles. In a previous study, Sharif et al. reported that gastric cancer cells were considerably more susceptible to oxidative stress generated by GaFe_2_O_4_@ Ag nanoparticles, which is in agreement with our finding^1^.

Flow cytometry analysis showed that treatment with BiFe_2_O_4_@Ag nanoparticles caused a significant increase in cell apoptosis and necrosis. However, the highest increase was observed for the late apoptosis (38.5 folds) suggesting that apoptosis induction is the major anticancer mechanism of the nanoparticles in gastric cancer cells. Previous studies reported that treating various cancer cell lines with silver and bismuth nanoparticles can trigger apoptogenic pathways^11–47^. Generation of ROS molecules by nanoparticles can cause lipid peroxidation, damage to DNA molecules, and disintegrate mitochondrial and cytoplasmic membranes which in response, triggers apoptotic pathways through the induction of the proapoptotic proteins as well as caspases^47^. Therefore, treating with BiFe_2_O_4_@Ag nanoparticles seems to initiate apoptosis pathways through the generation of oxidative stress and subsequent damage to the vital components of cancer cells.

Cell cycle analysis showed that treatment with BiFe_2_O_4_@Ag nanoparticles caused a significant increase in the population of the cells arrested at the S phase. The S phase is the stage before cell division during which the synthesis of DNA and histones occurs. Upon breakage of the DNA molecule, DNA replication will be stopped to activate DNA repair mechanisms. The activation of DNA repair mechanisms leads to a prolonged S phase to provide a longer time to repair DNA molecules^48^. Therefore, the increased population of the cells arrested at the S phase can be related to the cell chromosome damage through the generation of ROS molecules which may result in DNA breakage.

To investigate the possible nuclear damage in the nanoparticles-treated cancer cells, a Hoechst staining assay was performed. The results indicated damage to the cell chromosome which is in agreement with the result from the cell cycle assay. There is a close association between DNA damage and apoptosis^49^. Following extensive damage to the cell's DNA and the inability of the repair systems to repair the damage, apoptotic pathway signals are activated, which initially inhibit cell proliferation and can activate the cascade of caspase proteins, which break down cell proteins and induce cell apoptosis^46^. Therefore, it seems that following damage to the cell's DNA by BiFe_2_O_4_@Ag nanoparticles, cell proliferation is initially inhibited, mainly at the S phase, and then, apoptotic pathways are triggered in gastric cancer cells. However, conducting additional studies, especially investigating the role of apoptosis signaling molecules, is a limitation of this study, which should be considered in future studies.

## Conclusions

In this work, BiFe_2_O_4_@Ag nanoparticles were synthesized biologically using the *S. obliquus* extract and their anticancer mechanism on a gastric cancer cell line was characterized. Our results showed that the nanoparticles can inhibit the proliferation of the cancer cells, leading to cell cycle arrest at the S phase, and apoptosis induction. This study represents the anticancer potency of BiFe_2_O_4_@Ag nanoparticles against gastric cancer. However, it is important to acknowledge the limitations of our study including the relevance of *in-vitro* findings to *in-vivo* settings, the optimization of dosage and toxicity profiles, and the assessment of nanoparticle stability and pharmacokinetics.

## Data Availability

All data generated or analyzed during this study are included in this published article.
